# The spectrum of cardiovascular effects of dobutamine - from healthy subjects to septic shock patients

**DOI:** 10.5935/0103-507X.20170068

**Published:** 2017

**Authors:** Arnaldo Dubin, Bernardo Lattanzio, Luis Gatti

**Affiliations:** 1 Cátedra de Farmacologia Aplicada, Facultad de Ciencias Médicas, Universidad Nacional de La Plata - Buenos Aires, Argentina.; 2 Servicio de Terapia Intensiva, Sanatorio Otamendi y Miroli - Buenos Aires, Argentina.

**Keywords:** Dobutamine, Sepsis, Shock, septic, Cardiac output, Blood pressure, Microcirculation, Dobutamina, Sepse, Choque séptico, Débito cardíaco, Pressão sanguínea, Microcirculação

## Abstract

Dobutamine is the inotrope most commonly used in septic shock patients to increase cardiac output and correct hypoperfusion. Although some experimental and clinical studies have shown that dobutamine can improve systemic and regional hemodynamics, other research has found that its effects are heterogenous and unpredictable. In this review, we analyze the pharmacodynamic properties of dobutamine and its physiologic effects. Our goal is to show that the effects of dobutamine might differ between healthy subjects, in experimental and clinical cardiac failure, in animal models and in patients with septic shock. We discuss evidence supporting the claim that dobutamine, in septic shock, frequently behaves as a chronotropic and vasodilatory drug, without evidence of inotropic action. Since the side effects are very common, and the therapeutic benefits are unclear, we suggest that dobutamine should be used cautiously in septic shock. Before a definitive therapeutic decision, the efficacy and tolerance of dobutamine should be assessed during a brief time with close monitoring of its positive and negative side effects.

## Abstract (pt)

A dobutamina é o inotrópico mais comumente utilizado em pacientes com choque séptico, com o objetivo de aumentar o débito cardíaco e corrigir a hipoperfusão. Embora alguns ensaios clínicos tenham demonstrado que a dobutamina pode melhorar a hemodinâmica sistêmica e regional, outras pesquisas identificaram que seus efeitos são heterogêneos e imprevisíveis. Nesta revisão, analisamos as propriedades farmacodinâmicas da dobutamina e seus efeitos fisiológicos. Nosso objetivo foi demonstrar que os efeitos da dobutamina podem diferir entre voluntários saudáveis, estudos experimentais e insuficiência cardíaca clínica, em modelos de estudo em animais e em pacientes com choque séptico. Discutimos as evidências que suportam a afirmativa de que a dobutamina utilizada no tratamento do choque séptico frequentemente se comporta como fármaco cronotrópico e vasodilatador, sem evidências de ação inotrópica. Como seus efeitos colaterais são muito comuns e os benefícios terapêuticos não são claros, sugerimos que ela deve ser utilizada com cautela no choque séptico. Antes de uma decisão terapêutica definitiva, a eficácia e a tolerabilidade da dobutamina devem ser avaliadas por um tempo curto com monitoramento estrito de seus efeitos positivos e efeitos colaterais negativos.

## INTRODUCTION

The main goal of resuscitation in septic shock is the normalization of tissue perfusion through the administration of fluids, vasopressors, and inotropes. Dobutamine is the first-line inotropic drug recommended by the Surviving Sepsis Campaign.^(1)^ According to these guidelines, a trial of dobutamine infusion, up to 20µg/kg/min, should be administered or added to the vasopressor (if in use) in the presence of (a) myocardial dysfunction, as suggested by elevated cardiac filling pressures and low cardiac output, or (b) ongoing signs of hypoperfusion, despite achieving adequate intravascular volume and adequate mean arterial pressure.

Nevertheless, the clinical evidence supporting the beneficial effects of dobutamine on outcomes in septic patients is rather limited. The recommendations are based mainly on the first randomized controlled trial of early goal-directed therapy, in which only 14% of the patients received dobutamine.^(2)^ Moreover, some studies suggest that dobutamine has a low safety profile. A retrospective analysis of 420 patients with septic shock showed that the use of dobutamine was independently associated with increased 90-day mortality, even after adjustment, with a propensity score, for the inotropic treatment.^(3)^ In patients with severe heart failure, a meta-analysis also showed a trend towards worse outcomes with its use.^(4)^ Moreover, some observational studies found that dobutamine might cause variable hemodynamics effects and frequent side effects.^(5-7)^

Although dobutamine has been used for four decades in the treatment of septic shock,^(8)^ its pharmacodynamics is not well understood. The purpose of this review is to revisit some aspects of its clinical pharmacology. Our goal is to show that the effects of dobutamine are frequently unpredictable, heterogeneous, and dependent on the underlying condition.

## EFFECTS OF DOBUTAMINE IN NORMAL SUBJECTS

**Figure 1:**
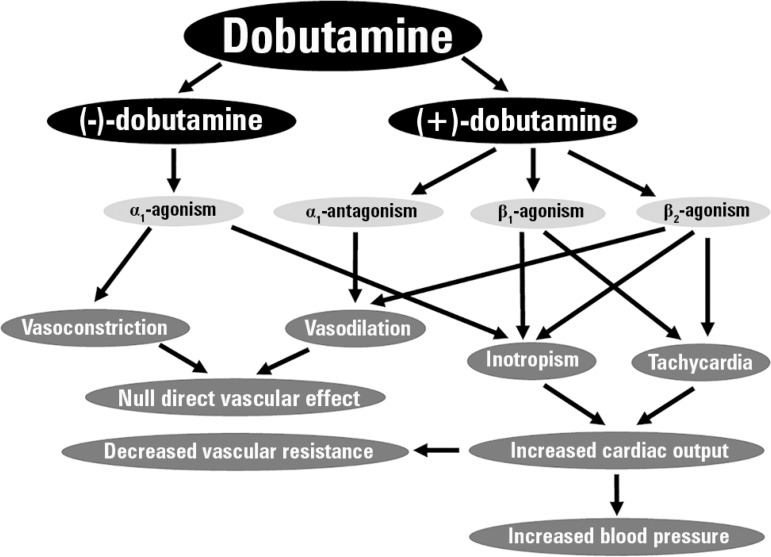
Schematic of the main pharmacodynamic and cardiovascular effects of dobutamine.

Dobutamine is used clinically as a racemic mixture.^(9-11)^ As such, the pharmacodynamic activity of the racemate will result from the interaction of the individual properties of the stereoisomers. (-)-dobutamine is a powerful adrenergic α_1_ agonist with weak β_1_ and β_2_ activity. In contrast, (+)-dobutamine predominantly stimulates β_1_ and β_2_ adrenoreceptors and exhibits α_1_ antagonist activity. Consequently, (±)-dobutamine mainly behaves as an inotrope. Since a significant part of the inotropic effect is related to increased cardiac α_1_ activity, dobutamine results in less tachycardia than other adrenergic drugs. In addition, there is no direct effect on vascular tone because of the opposing effects of each enantiomer: (-)-dobutamine is a vasoconstrictor, and (+)-dobutamine is a vasodilator. Accordingly, the decreases in vascular tone and peripheral resistance are adaptive adjustments to the simultaneous increases in cardiac output. Figure 1 summarizes the main pharmacodynamic and cardiovascular effects of dobutamine.

In healthy animals, dobutamine primarily increases stroke volume and cardiac output because of its inotropic effects. There is also a small increase in heart rate that is a minor contribution to the increase in cardiac output. The increased cardiac output induces an elevation in blood pressure and a reflex decrease in systemic vascular resistance.^(11,12)^

In healthy volunteers, increasing the dose of dobutamine results in elevated cardiac output, which is linearly related to its plasma concentrations.^(13)^ The relationships between heart rate and stroke volume to plasma concentrations, however, are more complex. Heart rate initially remains unchanged and then increases with higher concentrations, while stroke volume only increases at lower concentrations. Thus, cardiac output and blood pressure are linearly related to increasing doses of dobutamine. With 2.5µg/kg/min, both variables increase because of improved stroke volume. Further increases with higher doses only depend on tachycardia.

Beyond its hemodynamic effects, dobutamine produces a marked calorigenic effect, which is the consequence of complex α and β adrenergic metabolic effects. An infusion of 10µg/kg/min induces a rise in energy expenditure of 33% and a decrease in respiratory quotient secondary to increased fat metabolism.^(14)^

## EFFECTS OF DOBUTAMINE IN CARDIAC FAILURE

In some experimental models of cardiogenic shock, the actions of dobutamine are similar to those described in healthy subjects: increased cardiac output and blood pressure. In dogs with reduced cardiac contractility, low cardiac output, and hypotension, dobutamine produces dose-related increases in cardiac contractility and output, restores arterial blood pressure, and reduces total peripheral resistance.^(15)^

In patients with different forms of cardiac failure, however, the effects are not the same.^(16-18)^ Despite cardiac output improving, blood pressure does not increase. This finding suggests an unexpected primary effect on vascular tone and peripheral resistance not observed in either healthy subjects or models of experimental cardiogenic shock. In these circumstances, the augmentation in cardiac output goes together with elevations in blood pressure.^(11-13)^

## EFFECTS OF DOBUTAMINE IN SEPTIC SHOCK

In septic shock, dobutamine is typically used to increase cardiac output and oxygen transport. Since dobutamine has a thermogenic effect,^(14)^ it has also been used to evaluate the metabolic state of critically ill patients-the so-called dobutamine oxygen flux test. Some clinical studies found that the lack of increase in oxygen consumption in response to dobutamine is associated with worse outcomes, as an indication of a severe underlying cell disorder.^(19,20)^ Unfortunately, the clinical usefulness of this assessment has not been adequately established.

Even though dobutamine is recommended in septic shock to improve cardiac output and correct hypoperfusion,^(1)^ there are controversial reports about its effects in this setting. Several experimental and clinical studies showed beneficial effects on systemic hemodynamics.^(21-24)^ Moreover, increases in splanchnic perfusion and tissue oxygenation have also been found.^(25-29)^ A study performed in patients with septic shock showed that dobutamine increased oxygen transport, together with improvements in intramucosal acidosis and hyperlactatemia.^(27)^ In addition, dobutamine might be useful for the recruitment of microcirculation. In endotoxemic rats, dobutamine prevented the development of arteriolar constriction and maintained villus blood flow.^(30)^ Similar results were described in the hepatic sinusoidal microcirculation.^(31)^ In patients with septic shock, an infusion of 5µg/kg/min for 2 hours improved sublingual microcirculation.^(32)^ Interestingly, the microvascular effects were not correlated with changes in blood pressure or cardiac output.

Nevertheless, some data suggest that the effects of dobutamine in septic shock are not so predictable. The inotropic effect could be blunted in sepsis. An experimental study showed that the inotropic, but not the chronotropic, effect was reduced after an endotoxin challenge.^(21)^

**Figure 2:**
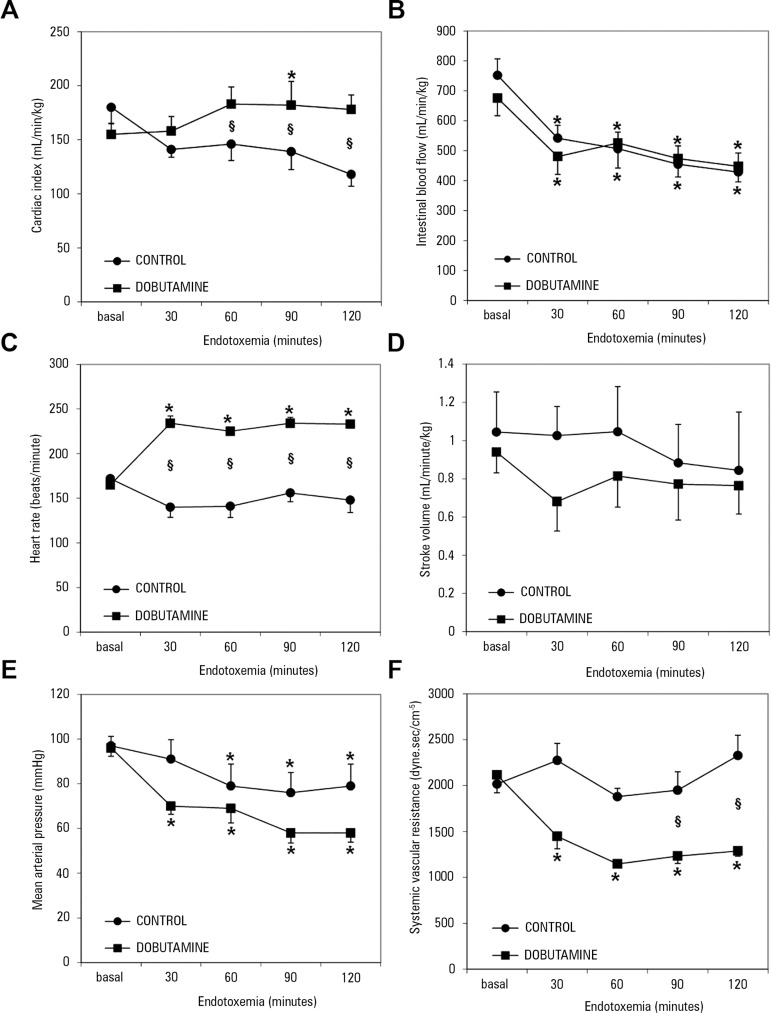
Behavior of hemodynamic variables in control and dobutamine-treated endotoxemic sheep. (A) Cardiac index; (B) superior mesenteric artery blood flow; (C) heart rate; (D) stroke volume; (E) Mean arterial pressure; (F) systemic vascular resistance.

Source: Modified from: Dubin A, Murias G, Sottile JP, Pozo MO, Barán M, Edul VS, et al. Effects of levosimendan and dobutamine in experimental acute endotoxemia: a preliminary controlled study. Intensive Care Med. 2007;33(3):485-94.^(33)^

A study performed in sheep endotoxemia showed that dobutamine increased cardiac output but decreased the fraction of blood flow directed to the gut.^(33)^ Moreover, the effect on cardiac output was completely explained by tachycardia, since the stroke volume was unchanged. In addition, there were decreases in blood pressure and systemic vascular resistance. The reduction in blood pressure and systemic vascular resistance, along with preserved cardiac output, imply that dobutamine primarily caused vasodilation (Figure 2). In this study, dobutamine induced vasodilation and tachycardia but did not show evidence of an inotropic effect. Furthermore, there was no improvement in gut perfusion. Similarly, in an experimental model of partial superior mesenteric artery occlusion, dobutamine, without fluid resuscitation, increased cardiac output but decreased the fraction of blood flow directed to the superior mesenteric artery and worsened intramucosal pH and the portal venous-arterial lactate gradient.^(34)^

In patients with septic shock, dobutamine might produce severe vasodilation. This phenomenon was evident in a randomized controlled trial in which 5 - 200µg/kg/min of dobutamine were used to reach supranormal values of oxygen transport. This therapeutic approach not only increased mortality, but larger doses of norepinephrine were also required in patients assigned to the dobutamine arm compared to the control group.^(35)^ Accordingly, maximal doses of norepinephrine were 1.20 *versus* 0.23µg/kg/min, respectively. In three large, randomized controlled trials in which dobutamine was used as part of the EGDT, no side effects related to dobutamine were reported.^(36-38)^ Nevertheless, the higher requirements for vasopressors in the EGDT group compared to the control group might reflect dobutamine-induced vasodilation.

Few clinical studies attempted to identify heterogeneous cardiovascular responses, taking into account individual responses. In these observational studies, the use of dobutamine was associated with erratic responses and frequent side effects.^(5-7)^ In one of these studies, 19 trials of increasing doses of dobutamine were carried out in 12 patients.^(5)^ In 12 cases, dobutamine was suspended because of hypotension or tachycardia. Most of the patients did not have increased stroke volume. Another study also showed unpredictable cardiovascular effects.^(6)^ Again, in most of the patients, dobutamine produced tachycardia and vasodilation without inotropic effects.

**Figure 3:**
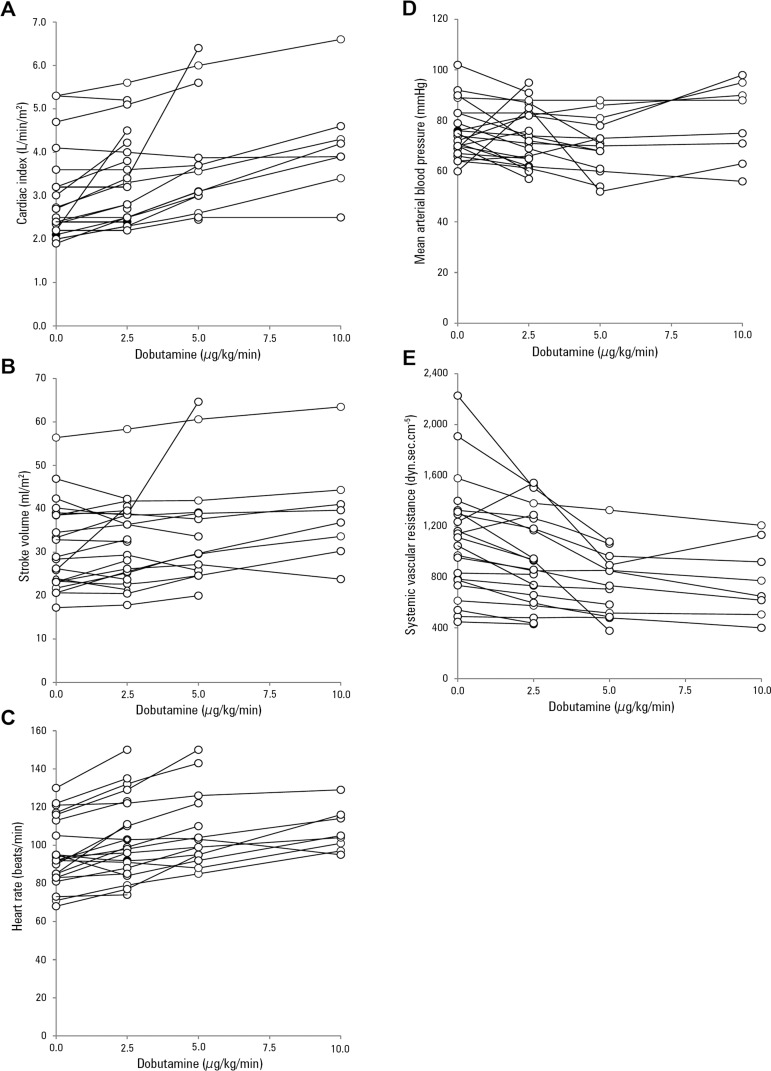
Individual behavior of hemodynamic variables in patients with septic shock treated with increasing doses of dobutamine. (A) Cardiac index; (B) stroke volume; (C) heart rate; (D) mean arterial pressure; (E) systemic vascular resistance.

Source: Modified from: Enrico C, Kanoore Edul VS, Vazquez AR, Pein MC, Pérez de la Hoz RA, Ince C, et al. Systemic and microcirculatory effects of dobutamine in patients with septic shock. J Crit Care. 2012;27(6):630-8.^(7)^

**Figure 4:**
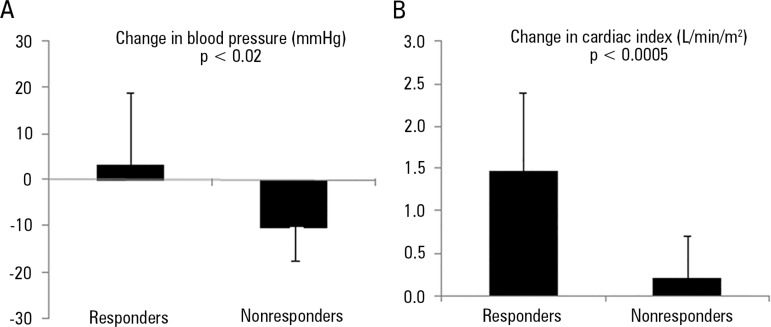
Difference in hemodynamic behavior of stroke volume-responder and non-responder patients with septic shock at the maximal dosage of dobutamine. In responders, dobutamine behaved as an inotrope, increasing blood pressure and cardiac index. In non-responders, dobutamine mainly acted as a vasodilator, since blood pressure decreased, and cardiac index marginally increased. (A) Change in mean arterial pressure; (B) change in cardiac index.

Source: Based on the publication data: Enrico C, Kanoore Edul VS, Vazquez AR, Pein MC, Pérez de la Hoz RA, Ince C, et al. Systemic and microcirculatory effects of dobutamine in patients with septic shock. J Crit Care. 2012;27(6):630-8.^(7)^

The third study assessed the effects of increasing doses of dobutamine in 23 patients with septic shock.^(7)^ The responses of hemodynamic variables to dobutamine were dichotomized according to changes greater than or less than 10% from baseline to the maximal dose. The maximal dosage of 10µg/kg/min was only reached in 8 patients. In most of the patients, the study could not be completed because of the occurrence of side effects, mainly decreases in blood pressure and increases in heart rate. Dobutamine increased cardiac output in 70% of the patients. There were opposite effects on mean arterial blood pressure: it decreased in 43% of the patients and increased in 22% of them. Stroke volume only improved in half of the patients. In most of the patients, the heart rate increased, and systemic vascular resistance decreased (Figure 3). There were no differences in baseline hemodynamics between patients with increased stroke volume in response to dobutamine and non-responders. Stroke volume responders, however, had lower left ventricle ejection fraction and more frequently showed systolic dysfunction and severe systolic dysfunction than non-responders did. Accordingly, the stroke volume changes in response to dobutamine correlated with baseline left ventricle ejection fraction. Stroke volume responders had a large increase in cardiac index and a trend toward increased blood pressure. In non-responders, cardiac output remained almost unchanged and blood pressure fell. These findings indicate that dobutamine behaved as an inotrope in responders, while it was only a vasodilator without inotropic effects in non-responders (Figure 4).

This study also evaluated the effects of dobutamine on sublingual microcirculation. In the group as a whole, there were no significant changes in microcirculatory variables. Nevertheless, there were variable individual responses. The behavior of the microcirculation was independent of systemic hemodynamics. The changes in perfused capillary density were not correlated with changes in either cardiac output or blood pressure. In contrast, changes were dependent on the basal state of the microcirculation. Therefore, patients with a compromised microcirculation at baseline had a positive response to dobutamine. On the other hand, a controlled crossover trial in patients with septic shock found detrimental effects of dobutamine on muscle and hepatic perfusion, a lack of improvement in peripheral perfusion, and a nonsignificant trend toward an increase in the proportion of perfused sublingual capillaries.^(39)^

The explanations for the heterogeneous responses to dobutamine in septic shock remain unclear. A putative reason is the inconstant pathophysiologic pattern of septic shock. The hemodynamic profile of septic shock results from interactions among variable components of hypovolemia, alterations in vascular tone, and myocardial dysfunction. After fluid resuscitation, most of the patients show hypotension, tachycardia, and normal or high cardiac output. Despite preserved systemic oxygen transport, septic shock patients frequently die from multiple organ failure or cardiovascular collapse. Typically, death from septic shock is related to the persistence of a hyperdynamic state with progressive and refractory vasodilation.^(40)^ Septic patients die from the inability to regulate peripheral circulation, not from low cardiac output. In this context, myocardial dysfunction may be a contributor to the hemodynamic instability but not the main cause. In addition, cardiac alterations in septic shock comprise systolic and diastolic dysfunction,^(41)^ dynamic left intraventricular obstruction,^(42)^ and acute stress cardiomyopathy.^(43)^ Diastolic dysfunction is common in septic patients (48%) and is related to increased mortality. In contrast, systolic dysfunction is less frequent (30%) and does not influence outcomes.^(41)^ Dynamic left intraventricular obstruction is found in 22% of patients with septic shock and is associated with poorer outcome.^(42)^ Only systolic dysfunction can be improved with the use of dobutamine; diastolic dysfunction, dynamic left intraventricular obstruction, and acute stress cardiomyopathy might be worsened. Therefore, dobutamine will not be useful for most of the cardiac alterations in septic shock. As supported by the results of an observational study,^(7)^ only patients with systolic dysfunction have positive responses in stroke volume. This observation might be an explanation for the heterogeneous responses to dobutamine.

Nevertheless, several other factors might contribute to the variability in responses. Sepsis is characterized by alterations in the adrenergic receptors, which subsequently may modify the response to catecholamines.^(44-46)^ Genetic polymorphisms might also be involved in the hemodynamic response. In healthy individuals, resting heart rate responses to 6µg/kg/min of dobutamine were 4.7-fold larger in Arg389Arg than in Gly389Gly homozygotes.^(47)^ Another study showed that dobutamine resulted in larger heart rate and contractility increases and diastolic blood pressure decreases in Arg389- *versus* Gly389-beta1AR subjects.^(48)^ On the other hand, it has been reported that polymorphisms do not substantially influence the magnitude of hemodynamic response to dobutamine during dobutamine stress echocardiography.^(49)^ In addition, a blunted inotropic, but preserved or increased chronotropic, response to dobutamine, has been associated with the aging process.^(50,51)^ The age-related decreases in contractile function in response to dobutamine might be explained by an inability to increase myocardial glucose utilization.^(51)^ An assessment, by means of cardiac magnetic resonance imaging, showed that dobutamine markedly decreased the passive left atrial emptying function and correspondingly increased the active emptying function in older adults (60 - 70 years) but not in young (20 - 30 years) healthy subjects.^(52)^ Accordingly, older adults had lower cardiac output during dobutamine stress. Finally, women might have an increased chronotropic response,^(53)^ but the data are inconclusive.^(54)^

## CONCLUSIONS

Patients with septic shock commonly display varying responses to dobutamine. Frequently, vasodilation and tachycardia are the most prominent effects without evidence of improved cardiac performance. These findings suggest a low profile of efficacy and safety. In addition, the effects on regional and microcirculatory perfusion are also unpredictable. The presence of echocardiographic systolic dysfunction and severe microvascular disorders could help in the identification of patients who would benefit from the use of dobutamine. Before a definitive therapeutic decision, efficacy of and tolerance to dobutamine should be evaluated during a brief time with close monitoring of its positive and negative effects.
